# Developing a novel prediction model in opioid overdose using machine learning; a pilot analytical study

**DOI:** 10.1002/hsr2.767

**Published:** 2022-08-08

**Authors:** Ehsan Sakhaee, Ali Amirahmadi, Morteza Mahdiani, Maziar Shojaei, Hossein Hassanian‐Moghaddam, Roman Bauer, Nasim Zamani, Hossein Pakdaman, Kourosh Gharagozli

**Affiliations:** ^1^ Brain Mapping Research Center, Department of Neurology Shahid Beheshti University of Medical Sciences Tehran Iran; ^2^ Department of Information Technology, School of Electrical and Computer Engineering, University College of Engineering Tehran University Tehran Iran; ^3^ Department of Artificial Intelligence ARIS Intelligent Solutions Company Tehran Iran; ^4^ Department of Computer Engineering Amirkabir University of Technology (Tehran Polytechnic) Tehran Iran; ^5^ Department of Clinical Toxicology, Loghman Hakim Hospital, School of Medicine Shahid Beheshti University of Medical Sciences Tehran Iran; ^6^ Social Determinants of Health Research Center, Department of Community Health Shahid Beheshti University of Medical Sciences Tehran Iran; ^7^ Department of Computer Science University of Surrey Guildford UK

**Keywords:** qEEG, opioid overdose, prognosis, mortality, machine learning, model fusion

## Abstract

**Background and Aims:**

The opioid epidemic has extended to many countries. Data regarding the accuracy of conventional prediction models including the Simplified Acute Physiologic Score (SAPS) II and acute physiology and chronic health evaluation (APACHE) II are scarce in opioid overdose cases. We evaluate the efficacy of adding quantitative electroencephalogram (qEEG) data to clinical and paraclinical data in the prediction of opioid overdose mortality using machine learning.

**Methods:**

In a prospective study, we collected clinical/paraclinical, and qEEG data of 32 opioid‐poisoned patients. After preprocessing and Fast Fourier Transform analysis, absolute power was computed. Also, SAPS II was calculated. Eventually, data analysis was performed using SAPS II as a benchmark at three levels to predict the patient's course in comparison with SAPS II. First, the qEEG data set was used alone, secondly, the combination of the clinical/paraclinical, SAPS II, qEEG datasets, and the SAPS II‐based model was included in the pool of classifier models.

**Results:**

Seven out of 32 (22%) died. SAPS II (cut‐off of 50.5) had a sensitivity/specificity/positive/negative predictive values of 85.7%, 84.0%, 60.0%, and 95.5% in predicting mortality, respectively. Adding majority voting on random forest with qEEG and clinical data, improved the model sensitivity, specificity, and positive and negative predictive values to 71.4%, 96%, 83.3%, and 92.3% (not significant). The model fusion level has 40% less prediction error.

**Conclusion:**

Considering the higher specificity and negative predictive value in our proposed model, it could predict survival much better than mortality. The model would constitute an indicator for better care of opioid poisoned patients in low resources settings, where intensive care unit beds are limited.

**Abbreviations**
ANN: artificial neural networks
APACHE: acute physiology and chronic health evaluation
aSAH: aneurysmal subarachnoid hemorrhage
DCI: Delayed Cerebral Ischemia
FLD: Fisher's linear discriminant
GCS: Glasgow coma scale
KNN: k‐nearest neighbors
LOO: Leave‐One‐Out
MCS: multiclassifier systems
MLP: multilayer perceptron
qEEG: quantitative electroencephalogram
SAPS: Simplified Acute Physiology Score
SVM: support vector machine

## 1 INTRODUCTION

Drug abuse, in particular, the abuse of heroin and morphine, is a global crisis and these two have been considered the drugs with one of the most potential adverse effects on human health.^1^ Data from the Centers for Disease Control and Prevention in 2020 suggests that Opioids were involved in 46,802 overdose deaths in 2018 (69.5% of all drug overdose deaths).^2^ In particular, methadone and tramadol have recently experienced increased exposure and therefore their associated mortality and morbidity have significantly increased.^3^, ^4^, ^5^, ^6^, ^7^, ^8^, ^9^

Altered levels of consciousness are common in these poisonings.^3^, ^4^, ^8^, ^9^ Intoxicated patients may be referred with unstable vital signs, and their severity scores are generally higher than other patients at presentation; however, they usually improve easier with proper treatment and their prognosis may not be as severe as it appears on presentation.^10^ In fact, it is not clear whether scores such as acute physiology and chronic health evaluation II (APACHE II) can be used in poisoned patients to the extent they are used for general intensive care unit (ICU) patients.^10^

In recent years, attempts have been made to use quantitative electroencephalogram (qEEG) recordings as a predictor. Rots et al. used qEEG for the early detection of delayed cerebral ischemia (DCI) in aneurysmal subarachnoid hemorrhage (aSAH) and concluded that the implementation of qEEG for aSAH patients likely improves the early detection of DCI.^11^ Crepeau et al. in a study to determine the prognostic value of EEG in therapeutic hypothermia after cardiac arrest, concluded that certain EEG changes correlated with the outcome.^12^ In another study, Arzabou and colleagues mentioned that EEG‐related features could be used as predictors of septic ICU mortality and delirium.^13^ Hirsch (2004) reported that seizures after intracranial hemorrhage (ICH; mainly nonconvulsive) were accompanied by a remarkable increase in mass effect and a poor prognosis.^14^ However, there is no information on the prognostic value of EEG in drug poisoning patients.

Based on the recent advances in machine learning, medical researchers have tried this approach in the field of EEG.^15^ In a study conducted by Fingelkurts and colleagues, they confirmed the prognostic value of qEEG with regard to survival in vegetative and minimally conscious state patients.^16^ In another study carried out by Khodayari‐Rostamabad et al., the authors successfully combined pretreatment EEG data and machine learning to predict schizophrenia patients' response to clozapine therapy.^17^ Löfhede and colleagues applied Fisher's linear discriminant (FLD), support vector machine (SVM), and feed‐forward artificial neural networks (ANN) on burst‐suppression EEG from infants with perinatal asphyxia where SVM demonstrated better results compared to other methods.^18^ Tenev and associates showed that SVM could be used to distinguish adults with attention deficit hyperactivity disorder (ADHD) based on the EEG power spectrum.^19^

Currently, there is no computational method to determine the prognosis in opioid‐poisoned patients. However, the opportunity to identify patients at high risk of death would allow for the well‐informed direction of resources, and so may help decrease mortality in emergency departments. Moreover, EEG measurements are not costly and can be done at the bedside, so it is a practical tool in a clinical setting. Here, we evaluate the efficacy of qEEG data alone and fuse it with clinical/paraclinical data and Simplified Acute Physiology Score (SAPS) II (SAPS II scores consist of 17 variables including 12 physiologic factors, age, type of admission, and 3 variables regarding underlying diseases) in distinct scenarios for developing prediction models that differentiate surviving from nonsurviving opioid‐overdosed patients using machine learning. This approach may also help a better understanding of the underlying origins determining survival and nonsurvival, which can give rise to novel treatment options in the future.

## 2 MATERIALS AND METHODS

### 2.1 Study design and participants

This pilot prospective analytical study evaluated 32 opioid‐poisoned (opium, tramadol, methadone) patients admitted consecutively to a referral clinical toxicology ICU. Patients with the following conditions in the emergency room (ER) were recruited: (a) patients with a Glasgow coma scale (GCS) below 15 who reported a drug overdose and signs of opioid overdose including respiratory depression and miosis and (b) patients with unknown drug poisoning who had positive blood or urine tests for opioids.

Opioid overdose was suspected when signs and symptoms of opioid toxicity were observed. Confirmation was achieved using laboratory confirmatory testing, response to antidote (naloxone), and patient interview.

Patients who had undergone cardiopulmonary resuscitation (CPR), had no corneal reflex and no doll's eye in their primary examinations, denied opioid overdose or claimed multidrug poisoning after recovery (confirmed by negative urine test results), and those with sepsis, meningitis, and encephalitis during EEG recording and stroke or brain tumor in past medical history or brain computed tomography (CT) scans as well as pediatric patients and patients whose next of kin did not wish them to be included in the research were excluded before EEG recording.

Survivors were interviewed before discharge and confirmed the consumption of opioids. They also confirmed that they had no past history of meningitis, encephalitis, thyroid, and chronic liver dysfunction. In nonsurvivors, toxicology results during the autopsy were used for confirmation of diagnosis.

### 2.2 Clinical and laboratory variables

Clinical data including past medical history, past drug history, the occurrence of seizure from admission to EEG recording, the occurrence of seizure from EEG recording to discharge, vital signs, GCS, urine output, and fever during EEG were evaluated. Laboratory variables include arterial blood pH (pH), arterial blood CO_2_ pressure (pCO_2_), arterial blood O_2_ pressure (pO_2_), arterial blood bicarbonate (HCO_3_), serum creatinine (Cr), serum sodium (Na) and potassium (K), blood urea nitrogen (BUN), blood sugar (BS), bilirubin, complete blood count (CBC), as well as toxicology screening tests were recorded on presentation, in the second, third, fourth day after admission and also before EEG recording. These measurements were obtained in the lab exam and the abnormalities (in terms of clinical importance) were recorded in the first 24 h (after admission) and in the tests directly before EEG recordings. No missing data was observed to be handled.

### 2.3 Sedation

Nine (28%), two (6%), and one (3%) patient(s) had been sedated with combined midazolam (5–10 mg/h) and fentanyl (50–100 mg/h), midazolam alone (5 mg/h), and fentanyl alone (50 mg/h), respectively.

### 2.4 Brain imaging

Brain CT scans without contrast were performed for 29 patients based on on‐arrival clinical conditions and ER physician's decision. Based on the CT scans, one of the subjects had generalized white matter changes and two had generalized brain edema. These subjects were not excluded and survived.

### 2.5 EEG recording

Electroencephalograms were performed in an eye‐closed position with at least 10 min duration and using a portable EEG device (NCC System) placed on the scalp at the 10–20 international system coordinates. The electrode impedances were checked online and EEG signals were amplified and recorded with a sampling frequency of 128 Hz.

### 2.6 Data processing and analysis

Clinical and paraclinical data were compared between survivors and nonsurvivors by applying the student *t*‐test, Mann–Whitney *U* test if variables were normally/not normally distributed, respectively. To evaluate the association between survivors/nonsurvivors and other categorical variables, *χ* ^2^ or Fisher's Exact tests were used. All variables showing a significant correlation with survival/death in univariate analysis were also tested in multivariable analysis. In the regression model, we entered all variables with *p* values less than 0.05 in their univariate analysis to determine independent variables predicting survival/death using the SPSS Enter method. Receiver operating characteristic (ROC) curves were generated to test the ability of SAPS II, qEEG and fusion models (see below) in predicting survival/death with the highest simultaneous sensitivity, specificity, positive predictive value (PPV), negative predictive value (NPV), and accuracy using the SPSS software (version 22; SPSS Inc.).

The recorded EEG data was preprocessed including automatic artifact removal (eye blinks, eye‐rolling, muscular, and gross artifacts) by automatic editing with the option “very high sensitivity for Z score artifact rejection” and a selected duration of 30 min in the Neuroguide software version 2.8.1 (without change of other default values). Subsequently, the re‐referenced (to link ears montage), artifact‐removed EEG data was inputted to a Fast Fourier Transform analysis (FFT). The absolute power spectrum was then computed for each electrode by taking the absolute values obtained from the FFT. All processes were done using a standard pipeline in the Neuroguide software. Data from each data set were then transformed into vector format, with tag “0” for survivors and tag “1” for nonsurvivors. A random‐forest model was applied to the datasets for selecting the most important features. We tested the result of selecting different numbers of features and finally found that selecting 20 features is the most efficient option. The algorithms were implemented using the Scikit‐learn library in Python where we used 32 examples with 266 attributes for absolute power data and 15 attributes for clinical/paraclinical data. Also, the absolute power and clinical/paraclinical data were fused both at the feature and model levels as we mentioned in Section 2.8 and 2.9. The evaluations of all trained models in each level of fusion were done by the means of Leave‐One‐Out Cross Validation (LOOCV) and all of the evaluation metrics reported in this study are the results of LOOCV criteria. Our analysis has three phases. First, is the preprocessing phase. Second, is the feature selection phase with the random‐forest algorithm. Third, the data processing phase uses the multiclassifier systems (MCS) approach.^20^

The datasets were first preprocessed by eliminating the ID of patients, sorting clinical and absolute datasets in the same order, and encoding categorical and string features to numerical values. Before the processing phase, a random‐forest model was used to select the 20 most important features.

**Figure 1:**
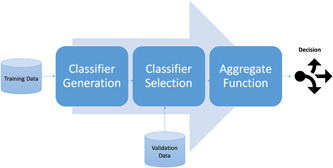
A multiple classifier system has three main steps. In the first phase, a collection of classifiers is generated (classifiers pool). Then, a set of classifiers is selected. In the end, the decision of the selected classifiers integrates and makes the final decision.^20^

In the processing phase, three levels of data analysis were devised to improve the mortality prediction of patients in comparison with SAPS II. The same processes were designed at all levels including (1) constructing a pool of classifiers (Including KNN, Decision Tree, Random Forest, Adaboost, MLP, SVM, logistic regression, and SAPS II), (2) selecting the best classifiers by their performance on the validation data set. (3) aggregating the selected classifiers' decisions and testing them (Figure 1). SAPS II is considered a benchmark (Table 1).

**Table 1:** Confusion matrix of different models among 32 opioid poisoned patients

Model	Prediction	Pred. false, *n* (%)	Pred. true, *n* (%)
SAPS II (Benchmark)	Actual false	21 (65)	4 (13)
	Actual true	1 (3)	6 (19)
Level 1^a^	Actual false	23 (72)	2 (6)
	Actual true	4 (13)	3 (9)
Level 2^a^	Actual false	23 (72)	2 (6)
	Actual true	4 (13)	3 (9)
Level 3^a^	Actual false	24 (78)	1 (3)
	Actual true	2 (6)	5 (13)

Abbreviation: SAPS: Simplified Acute Physiology Score.

^a^ The aggregated confusion matrix of the leave‐one‐out technique for: Level 1: qEEG data by Dynamic Ensemble Selection performance with dynamic frienemy pruning.

Level 2: Fusion of qEEG data and clinical/paraclinical data Overall Local Accuracy. Level 3: Majority voting on Random forest with qEEG data and clinical data and SAPS II classifiers.

### 2.7 qEEG data analysis

In this stage, the data set obtained from the EEG signals alone was used to predict the mortality of patients. As mentioned before, 20 important features were selected based on Random Forest feature importance criteria. Then MSC approach was implemented to predict the surviving and non‐surviving patients.

### 2.8 Feature fusion level

To achieve high prediction power, the idea of fusing the qEEG data set with gathered information for computing SAPS II was followed. At this level, we fused the qEEG data set with SAPS II and clinical/paraclinical data used in the SAPS II calculation. As for the other scenarios, the same procedure of creating a pool of classifiers and selecting the most efficient ones was utilized to analyze the fused data and obtain the optimized model.

### 2.9 Model fusion level

**Figure 2:**
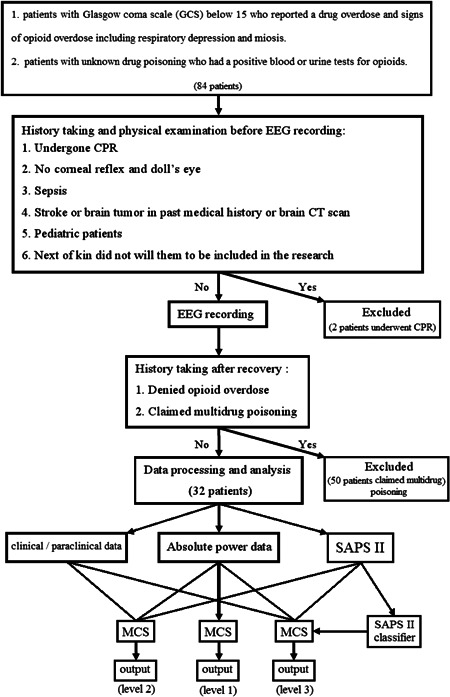
Schematic summary of the procedure for patient recruitment and prognosis prediction. Triple scenarios to assess the ability of qEEG data in mortality prediction in opioid overdose patients. In the first scenario, we enter qEEG data lonely to an MCS and evaluate it by LOOCV (level 1). In the second scenario, we enter the fusion of qEEG data and clinical/paraclinical data to MCS and assess the effect of this extra data in the final results (level 2). In the third scenario, input data are like the second scenario, but we push the SAPS II classifier in the classifier pool embedded in MCS (level 3). (In the schematic, “Yes” indicates that at least one of the conditions for exclusion was fulfilled.) LOOCV, leave‐one‐out cross‐validation; MCS, multiclassifier system; qEEG, quantitative electroencephalogram.

Since all resources of information were used, we planned to improve our prediction ability by adding a new model, obtained from SAPS II to our existing hyper model inferred from the fused data set. After appending the SAPS II model to the pool of classifier models and implementing the procedure of training, validating, and testing, we derived our final model. A summary of the methodological aspect of our work is shown in Figure 2.

### 2.10 Ethics approval and consent to participate

This study was approved by our local ethics committee at Shahid Beheshti University of Medical Sciences (IR.SBMU.SM.REC.1394.141) and has therefore been performed in accordance with the ethical standards laid down in the 1964 Declaration of Helsinki and its later amendments.

Informed written consent was taken from conscious patients. For loss of conscious patients who had no capacity for consent, it was taken from family members.

## 3 RESULTS

### 3.1 Clinical and paraclinical results

Seven out of 32 patients died. Table 2 shows the clinical and demographic information of the patients.

**Table 2:** Patient's characteristics (*n* = 32)

Age: median years (IQR)	40.5 (29.5–58.5)
Female, *n* (%)	4 (12.5)
Left hand, *n* (%)	1 (3)
Fever during EEG recording	10 (31)
Past medical history, *n* (%)	
Epilepsy	1 (3)
Cardiac disease	1 (3)
Depression	1 (3)
Schizophrenia	1 (3)
Chronic renal dysfunction	1 (3)
Convulsion in time from admission to EEG, *n* (%)	9 (28)
Convulsion in time EEG to discharge, *n* (%)	2 (6)
Time from admission to EEG: hours median (IQR)	28.5 (19–48.5)
Clinical status at time of EEG	
Not intubated n (GCS) 1(13)/1(10)/1(9)	
Mechanically ventilated, *n* (%)	
GCS 2–3	4 (12.5)
GCS 4–5	5 (16)
GCS 6–7	13 (41)
GCS 8–9	7 (22)
Length of hospitalization: days median (range)	8 (3–73)
Nonsurviving	17 (3–73)
Surviving	8 (3–21)
Poisoning	*n* (%)
Opium	9 (28)
Tramadol	5 (16)
Methadone	18 (56)
Outcome at hospital discharge, *n* (%)	
Nonsurviving	7 (22)
Surviving	25 (78)
SAPS II score mean	
Nonsurviving	52.7 ± 10.1
Surviving	40.8 ± 11.7

Abbreviations: IQR, interquartile range; SAPS, Simplified Acute Physiology Score.

**Figure 3:**
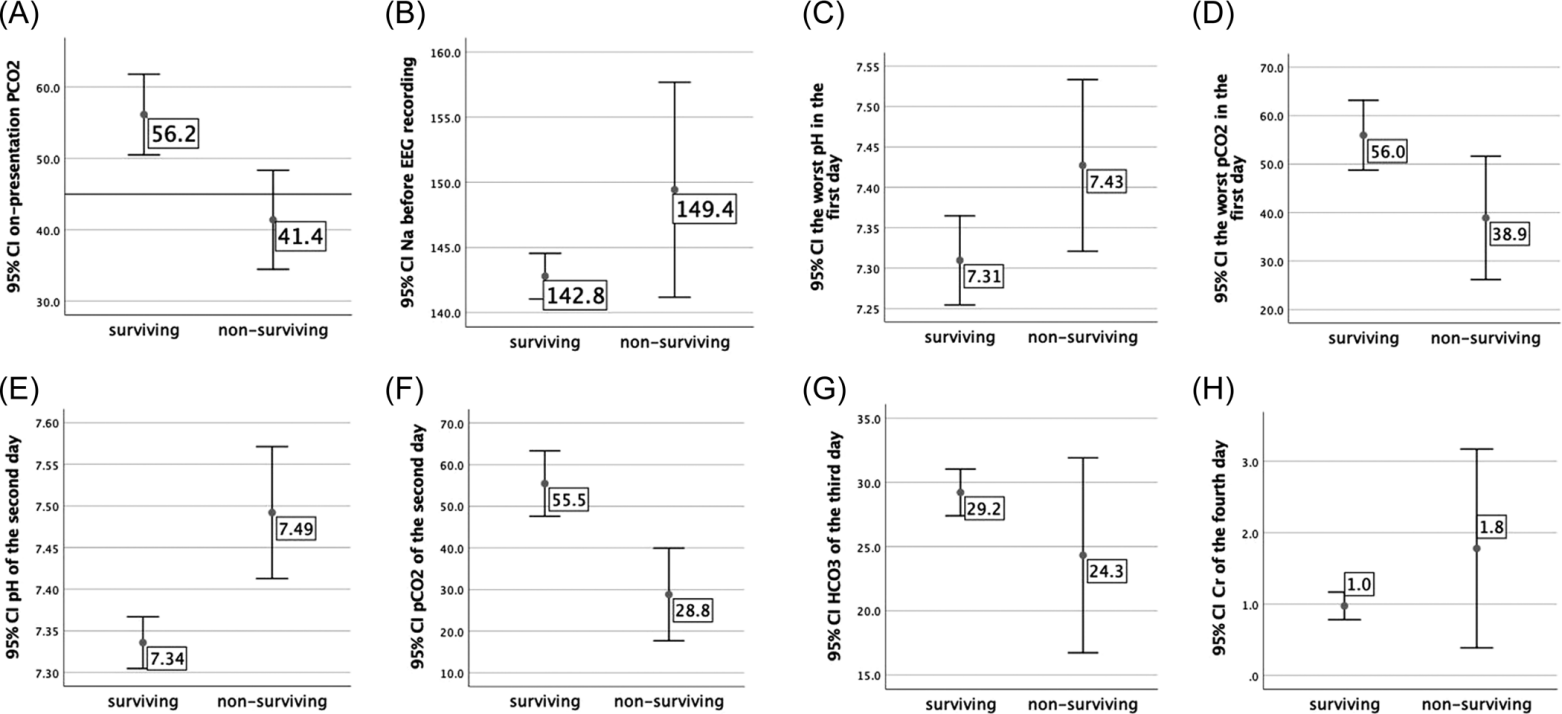
Distribution of variables that showed significant differences in univariate analysis with SPSS software (standard deviation is shown in error bars). (A) on presentation PCO_2_ measurements (B) measurements of Na before EEG recording (C) measurements of the lowest pH values on the first day (D) measurements of the highest PCO_2_ values on the first day E) pH measurements on the second day (F) PCO_2_ measurements on the second day (G) HCO_3_ measurements on the 3rd day (H) box plot of creatinine measurements on the 4th‐day postadmission.

On‐arrival and highest pCO_2_ and lowest pH values differed significantly between the two groups within the first 24 h. Also, the second‐day pH and pCO_2_ values and 3rd‐day bicarbonate values were significantly different between the two groups based on univariate analysis. Statistically significant results were found for the serum sodium levels before EEG recording and the 4th‐day creatinine levels. Regression analysis failed to find which of the abovementioned variables could predict death (Figure 3).

Mean (±SD) SAPS II were 41.1 ± 12.6 and 56.8 ± 6.2 in survivors and nonsurvivors, respectively (*p* = 0.02). SAPS II had a sensitivity of 85.7% and specificity of 84% for the prediction of mortality with a cut‐off of 50.5.

There were not any significant differences between the two groups in terms of brain CT scan findings and prescribing sedative drugs, so their effect on the EEG was not considered.

### 3.2 qEEG results

As the number of samples was too small, LOOCV was used to evaluate the models. In this cross‐validation analysis, the result of testing each sample was aggregated in a confusion matrix.

In the qEEG data analysis (level 1) and the feature fusion level (level 2), the results achieved by Dynamic Ensemble Selection performance with dynamic frienemy pruning^22^ (Supporting Information: Table 1) and data Overall Local Accuracy (Supporting Information: Table 1), respectively, yielded accuracy equal to 0.81, sensitivity equal to 0.42, specificity equal to 0.92 and F1 score equal to 0.90. In the model fusion level (level 3) the best result was obtained by majority voting including the generated classifiers in the classifiers pool and the SAPS II classifiers (Supporting Information: Table 1). This approach yielded an accuracy equal to 0.90, a sensitivity equal to 0.71, a specificity equal to 0.96, and an F1 score equal to 0.94. The best result was obtained when we improved the prediction model according to the model fusion level approach (described in section 2.6.3): the error rate decreased to 0.09 while in the first and second levels it was 0.15, so we were able to reduce the error rate by 40%. This means that by fusing the SAPS II model in level 3, we obtained an accurate predictor that had 40% fewer wrong decisions.

Table 3 shows diagnostic characteristics of four different models predicting mortality and survival in opioid poisoned patients.

**Table 3:** Diagnostic characteristics of four different models predicting mortality and survival in opioid poisoned patients.

Model	Sensitivity	Specificity	PPV	NPV	Accuracy
	(95% CI)	(95% CI)	(95% CI)	(95% CI)	(95% CI)
SAPS II	85.7	84.0	60.0	95.5	84.4
(Benchmark)	(42.1, 99.6)	(63.9, 95.5)	(36.8, 79.5)	(77.2, 99.2)	(67.2, 94.7)
Level 1	42.9	92.0	60.0	85.2	81.2
	(9.9, 81.6)	(74.0, 99.0)	(23.6, 87.9)	(75.0, 91.7)	(63.6, 92.8)
Level 2	42.9	92.0	60.0	85.2	81.2
	(9.9, 81.6)	(74.0, 99.0)	(23.6, 87.9)	(75.0, 91.7)	(63.6, 92.8)
Level 3	71.4	96.0	83.3	92.3	90.6
	(29.0, 99.3)	(79.6, 99.9)	(40.9, 97.3)	(78.8, 97.5)	(75.0, 98.0)

*Note*: *p* < 0.001: Ref to SAPS, Ref to 3, SAPS to 1, SAPS to 2, SAPS to 3, 1–2; *p* < 0.05: 1–3.

Abbreviations: NPV, negative predictive value; PPV, positive predictive value; SAPS, Simplified Acute Physiology Score.

## 4 DISCUSSION

The rate of opioid overdose deaths is increasing in many countries and we may need new prediction models to estimate mortalities.^21^, ^22^, ^23^, ^24^ A qEEG‐based machine learning approach alone was less accurate than SAPS II in predicting opioid poisoning mortality in our results. So, we tried to build a predictor model with the help of qEEG combined with clinical/paraclinical data (including SAPS II clinical parameters) as discussed in level 2. But the overall results did not differ. Moreover, the performance was similar to the performance when using the SAPS II‐based (traditional) method (Table 1). However, when we added SAPS II to level 2 as a model (i.e., level 3), the accuracy was greater than the SAPS II‐based approach. However, this difference was not statistically significant. So although our results show high diagnostic power, in particular in comparison with studies that had evaluated the power of scoring systems in the prediction of the outcome in poisoned patients, it has no advantage over SAPS II.^10^, ^25^, ^26^, ^27^, ^28^, ^29^ Such scoring systems are designed to quantify the severity of an illness, to develop quality control plans for patient care.

The higher specificity and NPV in different models can be explained by the fact that our model may help physicians identify patients that can survive if conservative management is done and they are appropriately taken care of. This finding is in accordance with previous studies that opioid‐related EEG abnormalities may be reversible, and normal qEEG may have a better prognosis factor for survival.^30^ APACHE II is calculated based on 12 physiologic criteria, age, and previous health state of the patient. To the best of our knowledge, our study is the first to attempt to use qEEG to predict mortality in opioid overdose. Alizadeh et al. examined the power of APACHE II and SAPS II in predicting mortality and morbidity rates in 195 patients hospitalized in toxicological ICU. Using an APACHE II score of more than 22 and using a SAPS II score of more than 59 yielded a sensitivity of 50% and 42%, respectively, for the prediction of mortality.^10^ The obtained specificity was 92% (in most of the studies on organophosphate poisoning, the sensitivity values were higher than specificity values). Other studies report very high sensitivity values (90%–100% for both SAPS II and APACHE II) while the specificity was between 60% and 90% for APACHE II and 70% and 80% for SAPS II.^26^, ^27^, ^31^, ^32^, ^33^ We would like to highlight that, although these studies are similar to ours in terms of sample size (23–48 patients), the type of toxicity is different. The scoring systems cannot precisely predict which patients will survive and have limitations including complicated calculations, the high number of variables, and uncertain measurements in various clinical conditions. Here we showed that fusing qEEG, clinical/paraclinical data, and SAPS II using machine learning can also be a valuable tool to predict survival.

In a study by Aghabiklooei and colleagues, clinical and laboratory factors were evaluated in methadone‐poisoned patients to predict their prognosis. Their results showed that the respiratory rate was significantly higher in nonsurvivors. They mentioned lower arterial blood pH and higher pCO_2_ as poor prognostic factors in their patients. Also, severe loss of consciousness and acute renal dysfunction were determined to be poor prognostic factors.^31^, ^34^

It should be borne in mind that the current study was done based on three common opioids in Iran. Some opioids like street fentanyl may not show up on a toxicology screen and the respiratory muscle paralysis that can occur seems more likely to cause brain injury than with traditional opioid agonists, where the major issue is respiratory depression, not apnea. Practically, clinicians and ICUs may seldom use any scoring system to change their management of individual patients. Moreover, even a perfect model may only have a small impact on the reduction of mortality rates, since in most cases the damage (if any) is done before the patient arrives in the hospital. Nevertheless, models are useful to provide objective information for family members and facilitate the discussion of goals of care. In cases where the survival rate is wrongly estimated, and with limited access to ICU beds, physicians in low and middle‐income countries may transfer these patients to a lower level of care to admit new patients in ICUs.

The patients were included with any level of loss of consciousness. This may be a potential problem limiting our results. Another limitation of the current study is its small sample size. Due to the limited number of patients, many static and dynamic classifier selections were employed for finding the best predictor and evaluated by LOOCV. By acquiring additional data in the future, the prediction performance of our proposed machine learning‐based approach may be further refined. There is also a significant sex imbalance in our study and due to limitations in the number of personnel, there was a waiting time between admission of some of the patients and their EEG recordings. Taking into account this time elapsed between drug use and EEG recording could be a further informative factor in future studies.

## 5 CONCLUSION

Although the machine learning was able to predict survival with more accuracy than SAPS using a combination of qEEG and clinical/paraclinical information, this difference was not significant in our study. This may support a prospective study utilizing qEEG to further strengthen the above results, and this can be the topic of a larger study.

## AUTHOR CONTRIBUTIONS

**Ehsan Sakhaee**: Data curation; Investigation. **Ali Amirahmadi**: Data curation; software; validation. **Morteza Mahdiani**: Data curation; software; Validation; writing—review and editing. **Maziar Shojaei**: Data curation; investigation; visualization. **Hossein Hassanian‐Moghaddam**: Conceptualization; formal analysis; writing—review and editing. **Roman Bauer**: Validation; writing—review and editing. **Nasim Zamani**: Writing—original draft; writing—review and editing. **Hossein Pakdaman**: Conceptualization; supervision. **Kourosh Gharagozli**: Conceptualization; supervision. All authors have read and approved the final version of the manuscript. Corresponding authors had full access to all of the data in this study and take complete responsibility for the integrity of the data and the accuracy of the data analysis.

## CONFLICT OF INTEREST

The authors declare no conflict of interest.

## TRANSPARENCY STATEMENT

H. H. M. and M. M. affirm that this manuscript is an honest, accurate, and transparent account of the study being reported; that no important aspects of the study have been omitted; and that any discrepancies from the study as planned (and, if relevant, registered) have been explained.

## Data Availability

The data that support the findings of this study are available on reasonable request from the corresponding authors.
